# Obstructive Sleep Apnea and Risk of Postoperative Complications after Non-Cardiac Surgery

**DOI:** 10.3390/jcm13092538

**Published:** 2024-04-26

**Authors:** Rabail Arif Chaudhry, Lori Zarmer, Kelly West, Frances Chung

**Affiliations:** 1 Department of Anesthesiology and Pain Medicine, Banner University Medical Center, University of Arizona COM-T, Tucson, AZ 85724, USA; 2 Memorial Hermann Hospital—TMC, Department of Anesthesiology and Critical Care Medicine, McGovern Medical School, University of Texas at Houston, Houston, TX 77030, USA; kelly.west@uth.tmc.edu; 3 University Health Network, Department of Anesthesiology and Pain Medicine, University of Toronto, Toronto, ON M5S 1A8, Canada; frances.chung@uhn.ca; 4 Institute of Medical Science, Temerty Faculty of Medicine, University of Toronto, Toronto, ON M5S 1A8, Canada

**Keywords:** obstructive sleep apnea, postoperative complications, perioperative evaluation, pulmonary risk factors, surgery, anesthesia

## Abstract

Obstructive sleep apnea (OSA), a common sleep disorder, poses significant challenges in perioperative management due to its complexity and multifactorial nature. With a global prevalence of approximately 22.6%, OSA often remains undiagnosed, and increases the risk of cardiac and respiratory postoperative complications. Preoperative screening has become essential in many institutions to identify patients at increased risk, and experts recommend proceeding with surgery in the absence of severe symptoms, albeit with heightened postoperative monitoring. Anesthetic and sedative agents exacerbate upper airway collapsibility and depress central respiratory activity, complicating intraoperative management, especially with neuromuscular blockade use. Additionally, OSA patients are particularly prone to opioid-induced respiratory depression, given their increased sensitivity to opioids and heightened pain perception. Thus, regional anesthesia and multimodal analgesia are strongly advocated to reduce perioperative complication risks. Postoperative care for OSA patients necessitates vigilant monitoring and tailored management strategies, such as supplemental oxygen and Positive Airway Pressure therapy, to minimize cardiorespiratory complications. Health care institutions are increasingly focusing on enhanced monitoring and resource allocation for patient safety. However, the rising prevalence of OSA, heterogeneity in disease severity, and lack of evidence for the efficacy of costly perioperative measures pose challenges. The development of effective screening and monitoring algorithms, alongside reliable risk predictors, is crucial for identifying OSA patients needing extended postoperative care. This review emphasizes a multidimensional approach in managing OSA patients throughout the perioperative period, aiming to optimize patient outcomes and minimize adverse outcomes.

## 1. Introduction

Obstructive sleep apnea (OSA) represents a common sleep disorder hallmarked by recurrent episodes of either partial or complete upper airway obstruction during sleep, leading to oxygen desaturation and sleep fragmentation. A confluence of variables influences this multifactorial collapse, including anatomical abnormalities (e.g., large tonsils, retrognathia), reduced muscle tone during rapid eye movement sleep, and neuromuscular factors such as inadequate compensatory responses to increased airway resistance [1,2]. These repeated obstructions result in a myriad of physiological responses, ranging from sympathetic activation to systemic inflammation, with potential repercussions on cardiovascular, metabolic, and pulmonary health [3].

With a global prevalence estimated at 22.6%, OSA has become a pressing health concern [4]. Within the surgical population, the prevalence of OSA varies between 7 and 10%, to nearly 70% among candidates for bariatric surgery [5]. Despite its widespread occurrence, affecting an estimated 24% of adult men and 9% of adult women, most OSA cases remain undiagnosed due to the subtle nature of symptoms and a lack of public awareness [6,7]. Empiric data indicate that patients diagnosed with OSA are at a heightened risk for postoperative complications such as cardiac adverse events, respiratory complications, significant oxygen desaturation, and an increased likelihood of intensive care unit (ICU) admission [8,9]. Particularly in non-cardiac surgeries, the unique physiological aberrations such as hypoxemia, enhanced sympathetic activity, and altered respiratory mechanics make OSA patients vulnerable to postoperative complications [10]. Furthermore, the effects of anesthesia, opioids, and surgical stress can exacerbate the perioperative risks in this patient population [11]. Despite these challenges, data regarding postoperative mortality within a 30-day window vary but generally indicate no significant increase. This phenomenon is speculated to be attributed to intensified postoperative patient monitoring. Therefore, it is imperative for optimal perioperative management to accurately identify these high-risk patients [9].

## 2. Preoperative Diagnosis and Screening

Remarkably, a vast majority of surgical patients with OSA remain undiagnosed, which has prompted the Society of Anesthesia and Sleep Medicine to advocate strongly for comprehensive OSA screening in the preoperative period [12]. Such screening helps make postoperative care safer by identifying patients that require extended postoperative monitoring or CPAP therapy. Anesthesiologists can tailor the anesthetic plan by avoiding sedatives or opioids, utilizing regional anesthesia techniques, and deciding if outpatient procedures are appropriate for these patients [13]. If possible, patients should ideally undergo an anesthesia preoperative evaluation a few weeks before their surgery for optimal management. The preoperative assessment should incorporate a medical history review, relevant screening questions, and a physical examination. Essential components of the review should cover concerns with the airway during previous anesthetics, hypertension, cardiovascular concerns, craniofacial abnormalities, and any previous sleep study findings. In addition to cardiopulmonary assessment, the physical examination must include a thorough assessment of the airway, neck size, tonsil size, and tongue volume.

Although sleep study testing remains the benchmark for OSA diagnosis, polysomnography (PSG) or home sleep apnea testing (HSAT) during the perioperative phase is neither economically practical nor logistically feasible. The costs, inconveniences, and potential scheduling conflicts associated with surgery make it challenging. The preoperative stage presents a window of opportunity to identify high-risk patients and subsequently refer them for a sleep study for OSA diagnosis and potential treatment. However, in situations where it is equally accessible to other modalities such as oximetry, HSAT would be preferable as it can both screen and diagnose. Various screening questionnaires have been formulated to assist in this crucial detection phase. While simple, cost-effective, and offering good sensitivity, these questionnaires are screening tools for OSA and are not for diagnostic purposes. Without a supplementary sleep study, their use can sometimes lead to misdiagnosis due to their varying specificity [12].

Validated questionnaires have emerged as a preferred modality for preoperative evaluation since they provide a quick, cost-effective means of risk stratification in diverse clinical settings. There are a number of validated screening tools for surgical patients such as the Berlin Questionnaire [14], the STOP-Bang questionnaire [15], the Perioperative Sleep Apnea Prediction Score (P-SAP) [16], and the DES-OSA score [17,18].

In order to be used in clinical practice, it is important for these screening tools to fulfill two primary objectives: (1) They should excel in accurately identifying patients with clinically significant OSA, defined by an AHI of 15 events per hour or greater (associated with an increased risk of cardiopulmonary disease and mortality), and (2) they should minimize the prevalence of false-positive diagnoses [19].

The STOP-Bang questionnaire has gained significant traction in diverse clinical environments. This eight-item tool, a blend of subjective and objective criteria, presents a graded risk assessment with each affirmative response, accumulating to a possible score of eight. A higher score corresponds with an escalated risk profile for OSA [20]. However, its specificity ranges between 30% and 43% when a score of three is used, leading to potential over-screening [15]. A systematic review and meta-analysis found the specificity increases to 79.4% when a threshold of five is used, suggesting that some of the limitations may be overcome by clinician familiarity with proper score interpretation. With the addition of a HCO_3_ level ≥ 28 mmol/L to the STOP-Bang score ≥ 3, the specificities for all OSA, moderate/severe OSA, and severe OSA improve to 85.2%, 81.7%, and 79.7%, respectively [21]. Therefore, testing for serum HCO_3_ level in a two-step screening process may be beneficial, especially in patients with coexisting obesity hypoventilation syndrome.

Overnight oximetry, which measures the oxygen desaturation index (ODI), significantly correlates with the AHI on PSG in surgical cohorts [22]. The ODI is defined as the number of (3% or 4% decrease in SpO_2_) desaturation episodes per hour. The ODI > 10 events per hour demonstrated a sensitivity of 93% and a specificity of 75% to detect moderate and severe OSA. In the surgical patient population, where preoperative recognition of OSA is critical for optimal perioperative management, the role of a nocturnal ODI from high-resolution pulse oximetry becomes pivotal. Chung et al. [22] demonstrated that various ODI thresholds have high sensitivity and specificity for predicting corresponding AHI levels. Notably, an ODI value less than or equal to 5 events per hour provided assurance in excluding moderate to severe OSA, while values greater or equal to 15 or 30 events per hour were indicative of moderate to severe, or severe OSA, respectively. A recent study utilizing samples from the Sleep Heart Health Study analyzed over 4800 patients using overnight in-home polysomnography with a pulse oximeter to track SpO_2_ levels during total sleep. Over a follow-up of approximately 10.7 years, 22.7% of participants died. Their results after adjusting for confounders indicated a significant association between average SpO_2_ levels during sleep and all-cause mortality, regardless of apnea–hypopnea index levels. Both maximum and minimum SpO_2_ levels during sleep were predictive of all-cause mortality, highlighting a significant association between nocturnal hypoxemia and mortality risk [23]. In summary, the ODI from pulse oximetry should be considered a valuable initial screening tool for OSA in surgical patients due to its high sensitivity and specificity. It offers a cost-effective and non-invasive alternative to PSG, helping healthcare providers identify patients who may require further diagnostic evaluation (those with an ODI > 15 events) and ensuring appropriate perioperative management.

**Figure 1:**
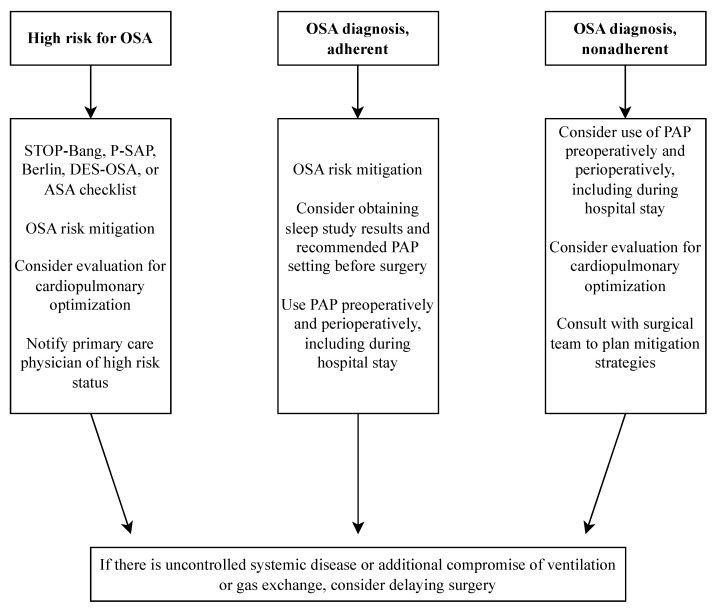
SASM Preoperative Guidelines for OSA [12]. Abbreviations: PAP, Positive Airway Pressure; P-SAP, Perioperative Sleep Apnea Prediction Score.

The Society of Anesthesia and Sleep Medicine endorses preoperative screening, reflecting a consensus on the potential benefits of identifying high-risk OSA patients before surgery [12] (Figure 1). This approach facilitates targeted perioperative precautions and interventions, which may contribute to reducing complications. The implementation of a preoperative OSA screening program carries significant public health implications, potentially improving the long-term health outcomes of patients through enhanced OSA management and reducing its associated health consequences. The hypothesis that preoperative diagnosis and optimization of OSA might yield benefits comparable to the perioperative management of chronic diseases like coronary artery disease and type 2 diabetes is gaining traction. Local healthcare settings should calibrate screening thresholds, weighing missed OSA diagnoses against total care costs. Lower thresholds improve sensitivity but increase resource use, while higher thresholds save resources but risk more missed diagnoses. In low-prevalence OSA populations, higher thresholds are recommended for resource efficiency. Most centers prefer using a higher STOP-Bang score of five or greater to indicate patients may be at risk of OSA. However, this area necessitates further research, particularly in developing screening tools that balance sensitivity with greater specificity than those currently available.

## 3. Decisions Regarding Proceeding with Surgery

In the decision-making process for surgical patients with OSA, multiple factors must be considered, and evidence-based generalizations are essential. Key considerations include specific comorbidities, the urgency of the surgery, types of surgery, need for high-dose opioids, and availability of monitoring [24,25]. Decisions on proceeding or delaying surgery should be individualized, involving the surgeon, perioperative providers, and the informed patient, especially regarding the increased risk of postoperative complications in untreated OSA. Risks include respiratory-related adverse events, such as reintubation and respiratory failure, which are potentially exacerbated by imbalances in pain processes and sensitivities to anesthetics or opioids [26].

Increasing evidence indicates that PAP therapy in OSA patients may help reduce postoperative complications [27,28]. A recent systematic review and meta-analysis found that in patients with OSA undergoing non-cardiac surgery, PAP therapy was associated with a 28% reduction in the risk of postoperative respiratory complications and a 56% reduction in unplanned ICU admission. In patients with OSA undergoing cardiac surgery, PAP therapy decreased the risk of postoperative cardiac complications and atrial fibrillation by 37% and 41%, respectively [29].

Consequently, the SASM guidelines highlight the importance of CPAP adherence as a key factor in determining the level of clinical vigilance and preoperative optimization required. Expert management of OSA by sleep medicine specialists has been shown to improve patient adherence to PAP therapy and reduce discontinuation of PAP therapy [30,31]. These specialists can address therapy refusal or poor adherence by implementing strategies to enhance acceptance.

Decisions regarding patient eligibility for ambulatory surgery are contingent upon several factors, including patient adherence to PAP therapy, the anticipated requirement for postoperative opioids, and the presence of other comorbid conditions. The Society of Anesthesia and Sleep Medicine Preoperative Guidelines recommend that additional evaluation and preoperative cardiopulmonary optimization should be considered in patients with diagnosed, partially treated/untreated, and suspected OSA if these three preexisting conditions are present: (1) hypoventilation syndromes, (2) severe pulmonary hypertension, and (3) resting hypoxemia in the absence of other cardiopulmonary disease [32]. The presence of OSA alongside acute or chronic cardiopulmonary conditions necessitates careful consideration, as such patients may not be suitable candidates for ambulatory surgery. In cases of untreated OSA, there may be benefits in delaying surgery to initiate and optimize PAP therapy not only to treat OSA but also to enhance postoperative respiratory function [27,28]. However, if delaying surgery is impractical, consideration should be given to inpatient admission for closer monitoring.

Patients with untreated OSA should be informed about their heightened risk of postoperative complications. It is advisable to have a low threshold for implementing postoperative oxygenation and ventilation monitoring in these cases [26]. The decision to extend such monitoring to an inpatient admission can be determined during an extended stay in the postanesthetic care unit [33].

## 4. Intraoperative Considerations

The use of anesthetic agents leads to dose-dependent depression of upper airway muscle function, which can prove particularly problematic in the context of OSA patients [34]. The main vulnerability is the attenuation in the activity of the pharyngeal dilator muscle, leading to a heightened risk of upper airway collapse. The primary site of occlusion, notably, is at the level of the soft palate during sleep [35]. The OSA condition worsens when sedated, especially in individuals with obesity. Multiple sites of airway collapse, complete airway obstruction, and incidents of hypoxemia are more common in these patients during procedural sedation. Anesthesia causes the upper airway to become even more collapsible than during natural sleep [36]. The implications of hypnotic agents, such as propofol, benzodiazepines, and inhalational drugs are profound, especially in OSA patients [37]. While integral to sedative processes, these drugs must be used judiciously in such patients due to the potential exacerbation of OSA symptoms, especially in contexts where the airway is not secured. The process of managing anesthetic depth is crucial. Implementing monitoring techniques like bispectral indexing or A-line autoregression can be invaluable. Such techniques guide the appropriate titration of hypnotic agents, potentially reducing the overall anesthetic doses required [38].

### 4.1. Neuromuscular Blocking Agents and Reversal

Neuromuscular blocking agents are commonly used for facilitating endotracheal intubation and ensuring muscle relaxation during surgical procedures. Nevertheless, these agents must be fully reversed. A notable occurrence of postoperative residual neuromuscular blockade exists, with potentially exacerbated consequences particularly in patients with OSA. Their obstructive airway is particularly vulnerable to collapse, leading to compromised upper airway function and subsequent complications, even when no respiratory symptoms exist [39]. There is compelling evidence indicating that these patients are at a considerably higher risk for postoperative complications linked to residual neuromuscular blockade. These complications include hypoxemia, respiratory failure, and other pulmonary complications [40]. Meticulous monitoring and confirmation of the complete reversal of neuromuscular blockade is essential in OSA patients. While both sugammadex and neostigmine are used for reversing neuromuscular blockades, the STRONGER trial found sugammadex to be superior with reduced risks for respiratory issues [41].

### 4.2. Anesthesia Techniques and Pain Management

Opioids are an essential component of pain control, but they pose pronounced risks for OSA patients, including an increased likelihood of opioid-induced respiratory depression [42]. These agents impair central respiratory mechanisms, weakening airway muscle tone and exacerbating episodes of obstructive apnea with compromised ventilation. Due to their inherent pathophysiology, patients with OSA have altered pain perception and an augmented opioid response [26]. This dual effect can lead to a significant reduction in postoperative opioid needs, putting undiagnosed patients at possible risk of receiving a potentially harmful dose. A prospective observational study utilizing polysomnography further strengthened the association between cumulative opioid consumption dose and postoperative severity of the AHI and central apneas [43]. Additionally, there is a significant association between postoperative naloxone requirement and OSA status [44]. Clinicians must be cautious when utilizing opioids in this high-risk population.

As clinicians navigate the intricacies of anesthetic choices for patients with OSA, an increasingly compelling argument is being made for the benefits of regional anesthesia over general anesthesia. In orthopedic surgery, neuraxial anesthesia has been shown to result in fewer complications when compared to general anesthesia [45]. General anesthesia was identified as a risk factor for hypoxemia in OSA, which predicted significant respiratory complications, ICU admissions, and increased length of hospital stay (LOS) [46]. In the first three postoperative nights, surgical patients with OSA experience a worsening of sleep-disordered breathing expressed as an increased AHI and exacerbation of nocturnal hypoxemia and hypercapnia [47]. Interestingly, general anesthesia was identified as an independent driver of an increased postoperative central sleep apnea index. In addition, the severity of the postoperative AHI was associated with 72 h cumulative opioid consumption in OSA patients [43].

**Figure 2:**
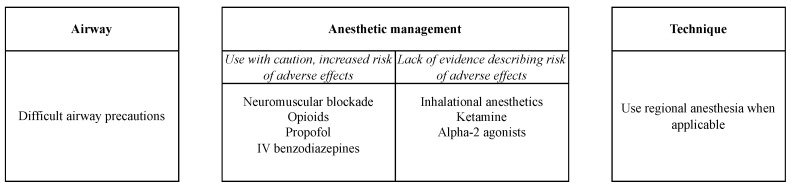
SASM Intraoperative Recommendations for OSA [50]. Abbreviations: IV, Intravenous.

Emerging research highlights the effectiveness of integrated, multimodal analgesia pathways encompassing various analgesic techniques (Figure 2). For patients with OSA, employing a combination of analgesic methods ranging from peripheral nerve blockade to acetaminophen, nonsteroidal anti-inflammatory drugs, cyclooxygenase-2 inhibitors, steroids, and ketamine was associated with opioid sparing and decreased critical complications. This impact was observed in a dose–response gradient, as increasing analgesic modalities were associated with stepwise beneficial outcomes related to reduced postoperative mechanical ventilation and ICU admissions [48]. Caution is advised when incorporating gabapentinoids into multimodal analgesia regimens due to their potential respiratory depression effects [49].

Post extubation, OSA patients exhibit an increased risk for complications, including rapid arterial oxygen desaturation and gastroesophageal reflux [51]. Proper patient positioning can mitigate some of these risks. Body positioning influences the upper airway’s collapsibility more than the sleep stages. Therefore, extubation and recovery should be in a lateral or semi-upright position to ensure optimal airway maintenance when possible.

In conclusion, the perioperative management of OSA patients requires a multidimensional approach, considering their unique physiological and pharmacological vulnerabilities. Tailored strategies that address anesthetic choice, pain management, and postoperative recovery can optimize outcomes and minimize complications.

## 5. Postoperative Management

### 5.1. Risk of Postoperative Complications

OSA has been linked with a heightened likelihood of various postoperative complications. These include cardiac and pulmonary issues, greater demands on hospital resources, and, possibly, mortality [52]. The existing literature shows that undiagnosed and untreated OSA poses a more significant risk of postoperative cardiovascular problems compared to those with treated OSA or without OSA, highlighting the criticality of preoperative identification and management of OSA in patients [13]. To date, the association of OSA with an increase in major postoperative cardiovascular or cerebrovascular events remains a subject of debate. A recent study analyzing the national inpatient sample indicated a significantly higher risk of postoperative major cardiovascular and cerebrovascular events across all surgical categories in patients with OSA [53]. This aligns with recent meta-analysis findings, which suggested that OSA is linked to an elevated risk of postoperative cardiopulmonary complications, postoperative delirium, bleeding, ICU admission, and extended hospital stays [54].

In the realm of the postoperative care of OSA patients, pulmonary complications require particular attention. The underlying pathophysiology of OSA, characterized by recurrent hypoxic events and airway instability, is a potent catalyst for a spectrum of respiratory complications. The incidence rate of any complication among OSA patients was reported at a compelling 48.9%, compared to 31.4% in patients without OSA. Of all complications, respiratory complications alone account for 40.4% versus 23.2% in controls [55]. Another comprehensive meta-analysis and trial sequential analysis also reported that OSA patients are nearly twice as likely to experience postoperative respiratory complications [56].

In a seminal study involving 337,333 non-cardiac orthopedic surgery cases, a diagnosis of OSA was strongly associated with prolonged hospital LOS and need for specialized postoperative care. OSA independently increases the risk of severe pulmonary events such as pulmonary embolism and Acute Respiratory Distress Syndrome, respiratory failure, and the necessity for ventilatory support [57]. Similarly, other studies have demonstrated an association between OSA and a higher incidence of oxygen desaturation, pneumonia, and respiratory failure despite adjusting for age and preexisting pulmonary issues [58]. Interestingly, despite the elevated risks, it has been reported that OSA patients exhibit similar rates of reintubation to controls, potentially pointing to the efficacy of focused supportive therapies [55,59]. Moreover, there was no marked difference in unplanned ICU admissions or hospital readmissions, indicating that targeted perioperative interventions can effectively manage these risks [56]. Both preoperative and postoperative screenings can identify these patients to optimize their postoperative management. It has been established that patients with positive preoperative screening for OSA have a significantly increased likelihood of having an oxygen desaturation index (ODI) > 10 events per hour in the first 24 h postoperatively [33]. Patients with recurrent Post-Anesthesia Care Unit (PACU) events were found to have a greater chance of experiencing ODI > 10 events per hour beyond the first 24 h, independent of screening results [33]. Consequently, these findings emphasize the importance of rigorous postoperative monitoring for OSA patients.

Currently, pulse oximetry stands as the predominant modality for postoperative respiratory surveillance. However, end-tidal carbon dioxide monitoring has demonstrated the capability to detect adverse respiratory events appreciably earlier prior to the manifestation of oxygen desaturation, even among patients on supplemental oxygen [60]. Given its enhanced sensitivity and specificity, capnography may become an integral instrument for increasing postoperative safety, presenting an early warning sign for detecting those patients most vulnerable to respiratory complications beyond the PACU environment. A systematic review and meta-analysis showed that continuous pulse oximetry after surgery increased the odds of recognizing desaturation by 15 times, and continuous capnography increased the odds of recognizing postoperative respiratory depression by 6 times [61]. Other innovative technologies, such as impedance-based non-invasive respiratory volume monitoring, have been introduced; however, their utility and practicality need further exploration [62].

**Figure 3:**
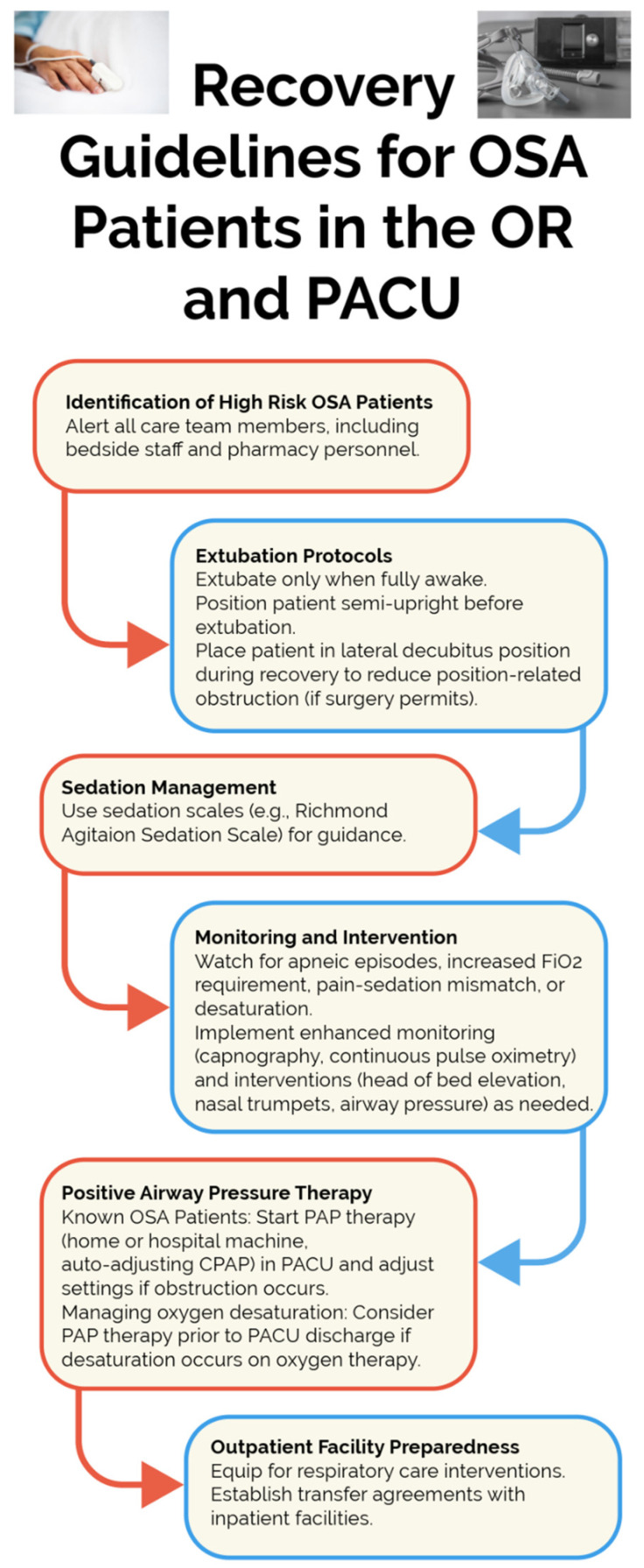
OSA Recovery Roadmap: Guidelines for the OR and PACU. Abbreviations: OR, operating room; PACU, postoperative anesthesia care unit; PAP, Positive Airway Pressure; FiO2, fraction of inhaled oxygen.

Despite the importance of vigilant postoperative respiratory and sedation oversight in OSA patients, the current literature does not sufficiently delineate the optimal timing for transition to unsupervised settings. This becomes particularly pressing considering the increasing literature that has identified preventable monitoring deficiencies as the primary catalyst for adverse outcomes in OSA patients [63,64]. The allocation of resources for postoperative surveillance of the expanding OSA cohort remains a significant concern for global healthcare systems. Figure 3 summarizes recovery guidelines for OSA patients in the OR and PACU. 

### 5.2. Supplemental Oxygen

Postoperative adjuvant oxygen administration in OSA patients has demonstrated enhanced oxygenation and a reduction in AHI without prolonging the apnea–hypopnea episode duration [65]. Based on the American Society of Anesthesiologists (ASA) guidelines, it is imperative to continuously administer supplemental oxygen (O_2_) to patients with OSA during their post-anesthesia recovery phase until patients demonstrate the capacity to sustain their baseline SpO_2_ on ambient air [66]. A particular cohort of OSA patients may exhibit carbon dioxide retention, attributable to the pivotal role of hypoxemia plays in precipitating respiratory arousals [65]. When supplemental O_2_ eradicates hypoxemia, there might be an elongation in the apnea duration culminating in hypoventilation and hypercarbia, thereby leading to decrease in respiratory arousal. This strategy aims to curtail hypoventilation, extended apneic phases, and atelectasis, which might go unnoticed by pulse oximetry in the context of O_2_ therapy, especially in the absence of supplementary monitoring mechanisms, such as respiratory rate tracking or capnography [66,67].In a recent systematic review and meta-analysis, O_2_ therapy is effective in reducing the apnea–hypopnea index in OSA patients and increasing SpO_2_. CPAP is more effective in reducing the apnea–hypopnea index than oxygen therapy, but O_2_ therapy and CPAP have similar effectiveness in increasing SpO_2_ and decreasing CT90. Also, high flow nasal O_2_ therapy is effective in reducing AHI [67].

### 5.3. Positive Airway Pressure

Positive Airway Pressure (PAP) therapy functions as a pneumatic stabilizer, targeting the prevention of airway collapse during sleep. Sustained PAP therapy has the potential to enhance the quality of life in OSA patients by improving ventilation, alertness, and cognitive function. Due to its often-perceived discomfort, adherence to PAP remains suboptimal, leading to uncertainties regarding its perioperative usage. In addition, the optimal duration for preoperative PAP therapy or the best time to commence PAP remain unknown. Consequently, it is reported that less than 20% of patients receive PAP therapy in the perioperative phase [68].

In addition, at present, there is a lack of robust evidence for the perioperative effectiveness of PAP therapy due to the impracticability associated with randomizing this widely accepted treatment, Nonetheless, a recent systematic review and meta-analysis [29] has provided valuable insights. For patients with OSA undergoing non-cardiac surgery, PAP therapy was associated with a significant 28% decrease in the risk of postoperative respiratory complications and a 56% reduction in the rate of unplanned ICU admissions.

In cardiac and thoracoabdominal surgeries, postoperative PAP application has been associated with reduced hypoxemia and decreased pulmonary complications, including reintubation [69,70]. Emerging yet limited evidence suggests that PAP utilization in OSA patients may mitigate postoperative major cardiovascular events, like cardiac arrest and cardiogenic shock [71]. When OSA patients were randomized to auto-titrated PAP preoperatively and for three days postoperatively, there was a significant decline in the AHI and improved oxygen saturation [72]. However, one study found that preoperative CPAP use did not significantly affect the total postoperative or respiratory complications [55]. Similarly, other studies report no significant reduction in postoperative complications [28,73]. Contrarily, the meta-analysis conducted by Berezin et al. highlights that, for patients with obstructive sleep apnea (OSA) undergoing cardiac surgery, PAP therapy significantly reduced postoperative cardiac complications by 37% and decreased the incidence of atrial fibrillation by 41% [29]. In light of the potential advantages of PAP without anticipated harm, both the ASA and SASM advocate for its preoperative implementation, especially in cases of severe OSA [66]. During surgery, the ASA advises the use of PAP or oral apparatuses for patients previously undergoing these treatments. Patients on PAP therapy should continue to use it both before and after surgery during their entire hospitalization. For previously untreated individuals, initiating PAP might be worth considering if there is severe OSA or if episodes of desaturation are detected postoperatively [66]. In addition, adaptive servo-ventilation, a form of bilevel Positive Airway Pressure therapy that is increasingly used to treat sleep-related breathing disorders, particularly central sleep apnea, may be utilized in postoperative OSA patients with significant uncontrolled hypoventilation. Table 1 outlines common contraindications to postoperative PAP. Table 2 presents the common types of PAP therapy for adults, along with their descriptions.

## 6. Critical Events and Mortality

The recent OSA Death and Near Miss Registry demonstrates that patients with OSA are particularly vulnerable to postoperative critical events, predominantly within the initial 24 h. Death and brain damage were more prevalent in scenarios involving unwitnessed events, the absence of supplemental oxygen, the lack of respiratory monitoring, and the use of a combination of opioids and sedative agents. Furthermore, PAP therapy, supplemental oxygen, and central respiratory monitoring, while beneficial, did not entirely eliminate the risk of catastrophic outcomes [75]. Case studies and malpractice reports frequently describe critical incidents or unexpected deaths in hospitalized patients with OSA, occurring even with proper narcotic dosing and initial alertness. A common pattern is patients being awake and stable initially but deteriorating or dying after falling asleep [76,77]. This has led to scrutiny of potential preventive measures, focusing on OSA subgroups exhibiting occult arousal failure, characterized by a delayed response to apneas and resulting in severe desaturation during sleep [76,78]. This issue is particularly elusive preoperatively as affected patients show no symptoms when awake, masking their risk within the surgical population. Hypnotics and narcotics exacerbate this risk by delaying arousal. The pathophysiology behind arousal failure in OSA, especially in severe or obese cases, is thought to involve a diminished response to arterial hypoxemia over time [76]. Currently, conventional screening methods do not effectively identify arousal failure perioperatively, although factors like obesity and disease severity may indicate a patient’s vulnerability. Table 3 summarizes postoperative considerations based on SASM consensus recommendations which could help mitigate such critical events and associated outcomes. 

## 7. Challenges and Future Research

Population-based studies reveal that the implementation of targeted interventions for OSA, such as regional anesthesia, supplemental oxygen, PAP therapy, and pulse oximetry monitoring remain underutilized. This is evidenced by reports from most anesthesiologists in North America indicating an absence of specific departmental protocols. The inconsistent practices in monitoring can be attributed to limited evidence supporting the effectiveness of these measures and the challenges posed by the rising prevalence of OSA, which strain the feasibility of implementing costly monitoring techniques [79]. Alarm fatigue and the discomfort caused by frequent false alarms add to these challenges [79].

Furthermore, the lack of comprehensive monitoring has been linked to severe outcomes in OSA patients [63,64]. The growing need for enhanced patient safety measures in the OSA patient population calls for the development of more refined screening and monitoring algorithms. These should be able to pinpoint specific OSA phenotypes at high risk of cardiorespiratory complications requiring intensive observation [79]. From a healthcare and economic standpoint, preventing complications is more advantageous than addressing them once they occur. Yet, the prevalent OSA screening methods are marred by high false-positive rates and insufficient specificity, leading to a misallocation of resources [79]. Identifying reliable risk indicators beyond the AHI, like SpO_2_, CO_2_ levels, pulse oximetry, and cardiac biomarkers, is crucial for improving patient safety in OSA [76,80]. Investigating novel methodologies is essential, given the complexity of early signs of clinical instability in OSA, which may not be discernible through a singular numeric threshold [76].

## Figures and Tables

**Table 1:** Contraindications to postoperative PAP [74].

Contraindications
- Agitated, uncooperative, or severely encephalopathic - Inability to protect the airway/excessive secretions or impending respiratory arrest - Signs of pneumothorax post laparoscopic abdominal surgery - Fixed upper airway obstruction or patients unable to tolerate the increased work of breathing (acute asthma, COPD) - Intracranial pressure > 20 mm Hg - Uncontrolled cardiac ischemia or arrhythmias or hemodynamic instability - Ongoing nausea/vomiting or hemoptysis or epistaxis - Severe facial deformities, for example, from reconstructive procedures or facial tumors - Facial burns - Esophageal surgery - Known or suspected tympanic membrane rupture or other middle ear pathology
Special Considerations:
- Comorbid neuromuscular disease * - Transsphenoidal surgery

* CPAP is not recommended due to increased breathing work on a weak diaphragm, while bilevel PAP devices without a backup respiratory rate can also fail. Advanced non-invasive ventilation modes that offer backup respiratory rates and enhance ventilation are beneficial, as they can aid rapid shallow breathing, improve gas exchange, allow respiratory muscle rest, and preserve respiratory muscle strength.

**Table 2:** Common types of Positive Airway Pressure (PAP) therapy [54].

Type of PAP Therapy	Description
Continuous Positive Airway Pressure (CPAP)	- Delivers a fixed, continuous level of pressure. - Typical pressure settings: 4 to 20 cm H_2_O. - Breathing effort is entirely from patient.
Auto-adjusting PAP (Auto-CPAP; Auto-BPAP)	- Provides a range of prescribed pressure (e.g., 5–20 cm H_2_O) automatically adjusted by the device based on a flow sensing algorithm. - Auto-CPAP adjusts in response to obstructive features. - BPAP and Auto-BPAP adjust to improve upper airway obstruction but require manual adjustments for hypoventilation; Volume-Assured Pressure Support (VAPS) is a preferred alternative. - Breathing effort is entirely from the patient.
Bilevel Positive Airway Pressure (BPAP-S; BPAP-ST)	- Delivers set Inspiratory PAP (IPAP, 8–30 cm H_2_O) and Expiratory PAP (EPAP, starting at 4 cm H_2_O). - Provides additional pressure support (IPAP-EPAP) to generate tidal volume. - Typically used for patients with restrictive and obstructive lung disease, or OSA patients intolerant to CPAP. - Can be with/without a backup respiratory rate. - “S” indicates spontaneous breathing, “ST” indicates spontaneous with timed backup respiratory rate.

**Table 3:** Postoperative Considerations (SASM Consensus Recommendations) [53].

Consideration	Details
After PACU Release	- Use overnight PAP therapy in hospitalized patients with known OSA. - Start empiric auto-CPAP for suspected OSA, especially if desaturations are noted. - Adjust PAP settings if obstructive events are observed. - Supplemental oxygen may be required but use cautiously. - Minimize postoperative nausea/vomiting with non-sedating anti-emetics. - Consider continuous capnography for monitoring hypercapnia. - Clinical judgment for additional respiratory monitoring: continuous pulse oximetry, capnography/end-tidal CO_2_, acoustic monitoring, placement in stepdown unit, telemetry.
Oxygen Therapy Considerations	- Oxygen may prolong apneas in some individuals. - Oxygen therapy can mask hypercapnia development. - Monitoring locations: ICU, stepdown units, general ward with additional monitoring capability. - Continuous pulse oximetry often recommended; respiratory rate monitoring as a surrogate. - Continuous capnography or transcutaneous CO_2_ monitoring may be useful.
Significant Respiratory Depression	- Immediate resuscitation with non-invasive positive-pressure ventilation, tracheal intubation, naloxone, or drug reversal agents. - Transfer ambulatory patients to inpatient facility for additional monitoring. - Place hospitalized patients in units experienced in treating OSA.
Postoperative Nights Consideration	- OSA severity may peak on postoperative night 3 when oxygen therapy is discontinued; cautious use of opioids and sedatives. - Provide appropriate follow-up care for patients with known and suspected sleep apnea.
High-Risk Patients	- Patients at high risk for OSA should follow up with a primary care provider or sleep specialist. - Identify patients at risk for OSA preoperatively to encourage postoperative evaluation.

## Abbreviations

OSA	Obstructive Sleep Apnea
SASM	Society of Anesthesia and Sleep Medicine
ASA	American Society of Anesthesiologists
AHI	Apnea–Hypopnea Index
ODI	Oxygen Desaturation Index
PSG	Polysomnography
